# Supplementary material for: Microbiological Characterization of Cutibacterium acnes Strains Isolated from Prosthetic Joint Infections
Source: Antibiotics (Basel). 2022 Sep 16;11(9):1260. doi: 10.3390/antibiotics11091260 (PMC9495218; doi:10.3390/antibiotics11091260)
Supplement: Supplementary file 1 [file antibiotics-11-01260-s001.zip › antibiotics-1897782-supplementary.pdf]

Table S1. Clinical data regarding PJI C. acnes strains included in this study.

Geographicalorigin	Strain	Phylotype	SLST	Joint site	Age*	Gender	Immunosuppressed
Slovenia	ESL1	H	IB	Hip	60	Female	No
Slovenia	ESL3	H	IB	Hip	60	Male	No
Slovenia	ESL4	D	IA	Hip	77	Male	Solid tumor
Slovenia	ESL5	H	IB	Knee	77	Male	No
Slovenia	ESL8	A	IA	Knee	59	Male	No
Slovenia	ESL9	H	IB	Hip	79	Male	No
Groningen, Netherlands	Hol 1	D	IA	Shoulder	76	Male	No
Groningen, Netherlands	Hol 2	D	IA	Shoulder	62	Male	No
Groningen, Netherlands	Hol 3	D	IA	Knee	79	Female	Hematologicalmalignancy
Groningen, Netherlands	Hol 6	D	IA	Hip	67	Female	No
Groningen, Netherlands	Hol 8	A	IA	Hip	65	Male	No
Groningen, Netherlands	Hol 9	D	IA	Knee	73	Male	No
Barcelona, Spain	BCN1	C	IA	Hip	60	Male	Autoimmunedisease
Barcelona, Spain	BCN2	A	IA	Hip	79	Female	No
Nantes, France	N1	A	IA	Shoulder	54	Male	No
Nantes, France	N2	H	IB	Hip	82	Male	No

Nantes, France	N3	H	IB	Hip	77	Female	No
Nantes, France	N4	D	IA	Shoulder	73	Male	No
Nantes, France	N5	A	IA	Shoulder	34	Male	No
Nantes, France	N6	K	II	Shoulder	64	Male	No
Nantes, France	N7	D	IA	Shoulder	74	Female	No
Nantes, France	N8	H	IB	Elbow	62	Male	No
Nantes, France	N9	D	IA	Shoulder	63	Male	No
Paris, France	P1	H	IB	Hip	45	Male	No
Paris, France	P3	K	II	Hip	76	Male	Unkown
Paris, France	P4	D	IA	Wrist	49	Male	Unkown
Paris, France	P5	H	IB	Shoulder	31	Male	No
Paris, France	P6	D	IA	Hip	58	Male	Unknown
Paris, France	P7	A	IA	Hip	72	Male	Solid tumor
Paris, France	P8	H	IB	Shoulder	46	Male	Solid tumor
Paris, France	P9	E	IA	Hip	25	Female	No
Paris, France	P10	C	IA	Hip	52	Female	No
Paris, France	P11	H	IB	Knee	26	Male	No
Paris, France	P12	K	II	Hip	25	Male	No

Paris, France	P14	D	IA	Shoulder	60	Male	No
Paris, France	P15	H	IB	Hip	79	Male	No
Paris, France	P16	A	IA	Knee	20	Male	Solid tumor
Paris, France	P17	A	IA	Shoulder	20	Female	Solid tumor
Paris, France	P19	K	II	Hip	39	Male	No
Paris, France	P20	F	IA	Hip	71	Male	No
Paris, France	P21	H	IB	Hip	81	Male	No
Paris, France	P23	D	IA	Hip	37	Male	No
Paris, France	P24	A	IA	Hip	75	Female	No
Paris, France	P25	F	IA	Knee	25	Male	Solid tumor
Paris, France	P26	K	II	Hip	57	Male	Unknown
Paris, France	P28	A	IA	Knee	43	Male	No
Paris, France	P29	K	II	Hip	50	Male	No
Paris, France	P30	H	IB	Hip	55	Male	Solid tumor
Paris, France	P31	H	IB	Shoulder	37	Male	No
Paris, France	P32	D	IA	Shoulder	46	Male	No
Paris, France	P33	K	II	Hip	75	Male	No
Paris, France	P34	H	IB	Hip	55	Male	No

Paris, France	P35	E	IA	Hip	35	Male	No
Paris, France	P37	H	IB	Shoulder	66	Male	No
Paris, France	P38	F	IA	Shoulder	70	Male	No
Zurich	ZH1	A	IA	Shoulder	81	Female	No
Zurich	ZH2	K	II	Shoulder	74	Male	Solid tumor
Zurich	ZH3	H	IB	Hip	76	Male	No
Zurich	ZH4	K	II	Hip	81	Male	No
Zurich	ZH5	H	IB	Shoulder	80	Male	No
Zurich	ZH6	H	IB	Shoulder	73	Male	No
Zurich	ZH7	H	IB	Hip	64	Male	No
Zurich	ZH8	C	IA	Hip	62	Female	No
Santander, Spain	S1	D	IA	Shoulder	70	Male	No
Santander, Spain	S2	K	II	Hip	76	Female	No
Santander, Spain	S3	H	IB	Shoulder	62	Male	No
Santander, Spain	S4	H	IB	Shoulder	44	Female	No
Santander, Spain	S5	E	IA	Hip	53	Male	Metotrexatetreatment
Santander, Spain	S6	A	IA	Shoulder	44	Female	No
Santander, Spain	S7	H	IB	Hip	76	Female	No

Santander, Spain	S8	A	IA	Shoulder	78	Male	No
Santander, Spain	S9	H	IB	Hip	74	Male	No
Santander, Spain	S10	A	IA	Hip	74	Male	No
Santander, Spain	S11	A	IA	Shoulder	60	Male	No
Santander, Spain	S12	D	IA	Knee	67	Female	No
Madrid, Spain	67	K	II	Hip	77	Female	No
Madrid, Spain	186	H	IB	Hip	76	Male	No
Madrid, Spain	213	D	IA	Knee	74	Female	No
Madrid, Spain	261	H	IB	Shoulder	53	Male	No

*Age at the time of infection.
